# Genome-wide gene expression profiling of the melon fly, *Zeugodacus cucurbitae*, during thirteen life stages

**DOI:** 10.1038/s41597-020-0387-9

**Published:** 2020-02-11

**Authors:** Dong Wei, Hui-Qian Xu, Dong Chen, Su-Yun Zhang, Wei-Jun Li, Guy Smagghe, Jin-Jun Wang

**Affiliations:** 1 Chongqing Key Laboratory of Entomology and Pest Control Engineering, College of Plant Protection, Southwest University, Chongqing, 400715 China; 2 International Joint Laboratory of China-Belgium on Sustainable Crop Pest Control, Academy of Agricultural Sciences, Southwest University, Chongqing, 400715 China; 3 State Cultivation Base of Crop Stress Biology for Southern Mountainous Land, Academy of Agricultural Sciences, Southwest University, Chongqing, 400715 China; 4 Department of Plants and Crops, Faculty of Bioscience Engineering, Ghent University, Ghent, 9000 Belgium

**Keywords:** Transcriptomics, Entomology, RNA sequencing, Agriculture

## Abstract

The melon fly, *Zeugodacus cucurbitae* (Coquillett), is an important destructive pest worldwide. Functional studies of the genes associated with development and reproduction during different life stages are limited in *Z. cucurbitae*. There have yet to be comprehensive transcriptomic resources for genetic and functional genomic studies to identify the molecular mechanisms related to its development and reproduction. In this study, we comprehensively sequenced the transcriptomes of four different developmental stages: egg, larva, pupa, and adults. Using the Illumina RNA-Seq technology, we constructed 52 libraries from 13 stages with four biological replicates in each and generated 435.61 Gb clean reads. We comprehensively characterized the transcriptomes with high-coverage mapping to the reference genome. A total of 13,760 genes were mapped to the reference genome, and another 4481 genes were characterized as new genes. Finally, 14,931 genes (81.85%) were functionally annotated against six annotation databases. This study provides the first comprehensive transcriptome data of all developmental stages of *Z. cucurbitae*, and will serve as a valuable resource for future genetic and functional studies.

## Abstract (summary)

**Table:** 

Measurement(s)	RNA • sequence_assembly • sequence feature annotation • gene expression
Technology Type(s)	RNA sequencing • sequence assembly process • sequence annotation • Expectation-Maximization(EM) algorithm
Factor Type(s)	biological replicate • developmental stage • sex
Sample Characteristic - Organism	Zeugodacus cucurbitae

Machine-accessible metadata file describing the reported data: 10.6084/m9.figshare.11698254

## Background & Summary

The melon fly, *Zeugodacus cucurbitae* (Coquillett) (Diptera, Tephritidae), is an important pest of cucurbit crops^1^. Although this species is thought to be native to India, it is now widely distributed in tropical, sub-tropical and temperate regions worldwide, including the Asia-Pacific and Africa^1,2^. *Z. cucurbitae* attacks more than 130 plant hosts from 39 families, among which the bitter gourd, snap melon and muskmelon are the preferred hosts^3^. Because of its powerful invasive ability, *Z. cucurbitae* has been defined as a category A fruit fly, with polyphagous, highly destructive characteristics^4^. Hence, although the *Z. cucurbitae* genome is available^5^, few functional genomic and genetic studies have been carried out comparing it to other species of tephritidae fruit flies. Functional studies of the key genes underlying mechanisms associated with *Z. cucurbitae* development, behavior, and reproduction are limited. Gene expression during development, which are useful for various analyses, have yet to be characterized.

Transcriptome sequencing is an efficient and low-cost method by which to explore gene expression patterns at multiple developmental stages^6,7^, tissues in insects^8,9^. Many insect transcriptomes have been obtained using next-generation sequencing techniques for functional genomic studies^10,11^. For example, in *Ceratitis capitata*, a conserved *Maleness-on-the-Y* gene was isolated to be involved in the male sex determination by RNA-Seq.^12^. Comprehensive studies of gene expression during various developmental stages in many important insect pests, e.g. the oriental fruit fly *Bactrocera dorsalis*^13,14^ and the ladybird *Henosepilachna vigintioctopunctata*^6^, have been performed to explore the molecular mechanisms underlying metamorphic development. In *C. capitata*, the gene expression at the specific embryogenesis stage was also obtained by transcriptomic sequencing technology^15^. In addition, gene expression has been studied in the fat bodies, ovaries, and testes of *B. dorsalis* to identify critical genes involved in female and male gonad-specific roles^8,16,17^. Comparative transcriptomic analyses have also been used to characterize the differential expression of genes putatively associated with fecundity during the maturation of *B. dorsalis* and *C. capitata* adults^18,19^. Transcriptomes have also been sequenced to investigate the mechanisms of the insect response to environmental stressors, including insecticides^20^ and microbial infection^21,22^. Thus, transcriptome sequencing allows us to determine the gene expression mechanisms underlying certain biological functions and to identify potential targets associated with pest control.

Many transcriptomes have already been published and published in the National Center of Biotechnology Information (NCBI) Sequence Read Archive (SRA) database, which is the most popular repository of raw RNA-Seq data. For example, more than 1398 samples of 47 projects have been published and open accessible for another insect pest species in the Tephritidae pest, *B. dorsalis*. However, for *Z. cucurbitae*, only 23 samples from five projects have been released (before August, 2019), and four of the five projects were sequenced for genome annotation and provided a fundamental reference regarding protein-coding genes of *Z. cucurbitae*^5,23^, but no more direct gene expression was exhibited, and no more biological replicates were performed. The remaining project, from 2016, was sequenced only from middle-aged adults and focused on proteins associated with chemosensory perception^24^, in which a small number (13) of odorant binding proteins (OBPs) were identified. In homologous *B. dorsalis* fruit fly, 49 OBPs were identified and characterized^25^. There is considerable gene expression information left to collect. The fruit fly is a holometabolous insect, and its life cycle has four developmental stages. In addition to the differences gene expression across these stages, complex biochemical processes take place within the larval development, pupal development, and sex maturation during the adult stage. For example, in larval development, the juvenile hormone and ecdysone are the most important effectors, and they cooperate with other genes and pathways to regulate the molting process^26,27^. Comparative transcriptomes were performed within the adult stage to explore the candidate genes involved in fecundity of *B. dorsalis*^18,19^. Complex biochemical processes also take place during the pupa stage in which the tissues were remodeled, e.g. SP95 involved in integument remodeling^28^. Gene expression during the specific time strictly in the process of the molting from old larva to the early pupa was also critical to successful molting, e.g. the wondering stage^14^. Although four transcriptomes were released, they are not a good resource because of the lack of biological replicates. The genome genetic sequence information is of great help for gene expression annotation, e.g. *C. capitata* and *Z. cucurbitae*^5,29^. A global gene expression profile for *Z. cucurbitae* has yet to be produced for assessment of gene expression throughout development are therefore still inaccessible.

**Fig. 1:**
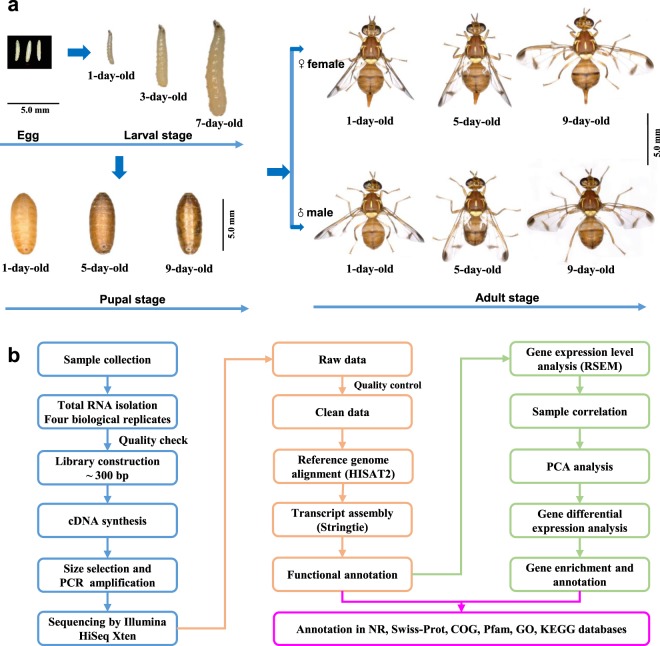
Schematic work flow of the transcriptomes from the sample preparation to sequencing. (**a**) Sample collection of *Zeugodacus cucurbitae*, and the 13 stages analyzed in this study are shown as photos. (**b**) Schematic overview of the sample collection, sequencing and analysis.

In this study, we performed the first deep transcriptomic sequencing of multiple different developmental stages of *Z. cucurbitae* by RNA-seq, including 13 stages, and 52 libraries (four biological replicates in each stage) (Fig. 1, Online-only Table 1). We generated a high-coverage dataset for genome-wide gene expression profiling. We successfully mapped 13,760 genes to the reference genome, and identified 4481 novel genes. In total, 14,931 genes (81.85%) were functionally annotated against the NCBI non- redundant (NR), Swiss-Prot, Pfam, Gene Ontology (GO), Cluster of Orthologous Group (COG), and Kyoto Encyclopedia of Genes and Genomes (KEGG) databases (Table 1). Four biological replicates of each stage provide a reliable evaluation of the gene expression during all the life stages. These data can also be used to analyze gene expression during metamorphosis to elucidate the molecular network, as well as the gene expression during the larval stages, pupal stage, and during the sexual maturation of adults. In addition, the comparative analysis of gene expression with other tephritidae fruit flies can also be performed, e.g. *B. dorsalis*, *C. capitata*, in which such data are available^14,30^. Our work represents a valuable resource, and will be helpful for future studies of functional genes associated with metamorphic development and reproduction.

**Table 1:** Gene assembly and database annotations.

Annotation	Transcripts		Genes		Percentage of annotated genes		
	Reference	New	Reference	New	Reference	New	Total
NR	21180	15261	12890	2001	93.68%	44.66%	81.63%
Swiss-Prot	16536	10522	9703	513	70.52%	11.45%	56.01%
COG	19961	13503	11960	1323	86.92%	29.52%	72.82%
Pfam	17852	11333	10653	645	77.42%	14.39%	61.94%
GO	10862	6464	6733	178	48.93%	3.97%	37.89%
KEGG	10863	6927	6333	320	46.02%	7.14%	36.47%
Total annotation	**21197**	**15337**	**12902**	**2029**	**93.76%**	**45.28%**	**81.85%**
Total	22159	19371	13760	4481	**100.00%**	**100.00%**	**100.00%**

## Methods

### Insect culture, sample collection

*Z. cucurbitae* were collected as pupae from Haikou, Hainan Province, China, in 2016, and were reared at 26–27 °C with 70 ± 5% relative humidity and a photoperiod of 14:10 h light: dark. Similar to *B. dorsalis* artificial diet^31^, *Z. cucurbitae* larvae were reared on an artificial diet consists of bitter gourd, corn, yeast powder, wheat flour, and sucrose; adults were fed a mixture of sucrose, yeast powder, and honey. Samples were collected at several different developmental stages: egg (~100 individuals < 8 h post-laying), 1^st^ instar larva (100 individuals at 1-d-old), 2^nd^ instar larva (20 individuals at 3-d-old), 3^rd^ instar larva (10 individuals at 7-d-old), pupa (three individuals at 1-, 5-, and 9-d-old, representing the early-, middle-, and late-stage of pupae respectively), virgin female and male adults (three individuals at 1-, 5-, and 9-d-old) (Fig. 1a). As preliminary experiments showed that adults were sexually mature at 9-day-old, the time points 1-, 5-, and 9-d-old represented the newly-emerged, vitellogenic, and sex-matured stages, respectively. Four biological replicates of each stage were prepared.

### Total RNA isolation

Total RNA was extracted from the 13 stages with TRIZol reagent (Invitrogen, Carlsbad, CA, USA), following the manufacturer’s instructions. The concentration and purity of total RNA samples were firstly quantified using a NanoDrop One (Thermo Fisher Scientific, Madison, WI, USA), and were re-determined using a Bioanalyser 2100 (Agilent Technologies, Palo Alto, CA, USA) before library construction.

### Library construction and sequencing

The transcriptome libraries were constructed using the TruSeq RNA sample preparation kit (Illumina, San Diego, CA, USA), following the standard instructions. In brief, all the mRNA was isolated using the polyA selection method with oligo (dT) magnetic beads (Illumina), and then fragmented using fragmentation buffer. Double-stranded cDNA was synthesized using a SuperScript double-stranded cDNA synthesis kit (Invitrogen, Carlsbad, CA, USA) with buffer, dNTPs, random hexamer primers, DNA polymerase I, and RNase H. The synthesized cDNA was subjected to end-repair, phosphorylation, and ‘A’ base addition following the Illumina library construction protocol. Each library was size selected for cDNA target fragments (200–300 bp) on 2% low range ultra-agarose gels, then PCR amplified using Phusion DNA polymerase (NEB, Ipswich, MA, USA). Finally, paired-end RNA sequencing libraries were sequenced with the Illumina HiSeq Xten (2 × 150 bp read length). The raw data files obtained from high-throughput sequencing were converted to the original sequence.

### Preprocessing of sequencing data

**Fig. 2:**
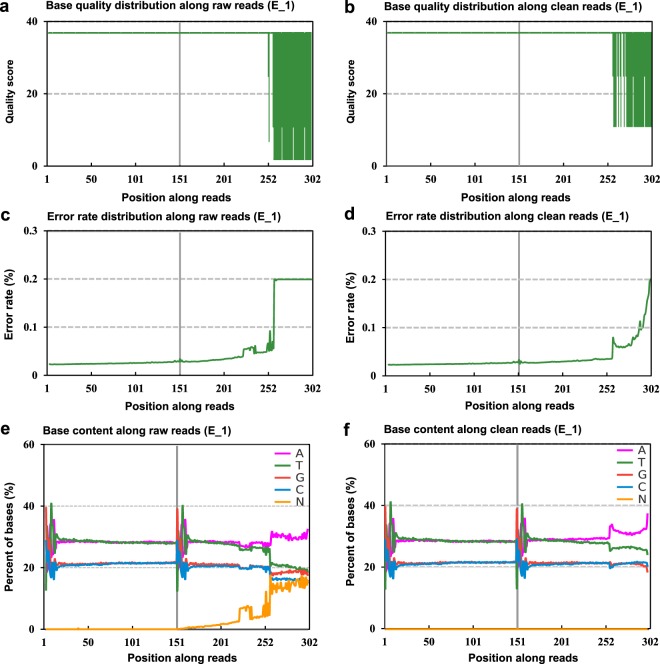
Quality assessment of the RNA-Seq. Example of the base quality of the raw reads (**a**) and clean reads (**b**). Error rate distribution of the raw reads (**c**) and clean reads (**d**). Base content of the raw reads (**e**) and clean reads (**f**).

We constructed 52 libraries and produced 3,013,315,500 clean reads (435,605,241,804 bp) (Online-only Table 1). The raw paired-end reads were trimmed and quality controlled using SeqPrep (https://github.com/jstjohn/SeqPrep) and Sickle (https://github.com/najoshi/sickle) with default parameters of the sequencing analyzer^32^. The quality of each base was evaluated by FASTX (Fig. 2a,b). The error bases with N and low quality reads were removed (Fig. 2c–f). Clean reads were aligned to the reference genome using HISAT2 (http://ccb.jhu.edu/software/hisat2/index.shtml)^33^. The mapping criteria were as follows: sequencing reads should be uniquely matched to the genome, with up to two mismatches allowed but without insertions or deletions. The mapping ratios were calculated as an index of the sequence quality. The quality assessment of the transcriptomic sequencing was evaluated using RSeQC 2.3.6 package. The highest coverage paths were assembled and constructed as transcript isoforms. All clean reads those were not mapping to the transcriptional regions were assembled into new transcripts based on overlaps using StringTie (http://ccb.jhu.edu/software/stringtie)^34^.

### Gene annotation

All genes, including the newly assembled fragments, were functional annotated against several databases (NR, Swiss-Prot, and evolutionary genealogy of genes: Non-supervised Orthologous Groups) using DIAMOND v0.8.37.99. For Pfam annotations, HMMER (version 3.1b2) was used with an *E*-value < 10^−5^. For Gene Ontology (GO) annotations, the Blast2GO pipeline was used, with an *E*-value < 10^−5^ ^35^. Pathway analysis was performed against the KEGG database with KOBAS v2.1.1 (*E*-value < 10^−5^). A total of 81.58% genes were annotated in six databases (Table 1).

### Gene expression analysis

Gene expression analysis was conducted based on the sequenced reads using RNA-Seq with the Expectation-Maximization (RSEM, v1.3.1) method^36^. Global gene expression was quantified as transcripts per million reads (TPM) in the four biological replicates. The average TPM value after normalization represented the corresponding quantitative gene expression level at each developmental stage^37^. The average TPM values for each stage were also used in a principal component analysis (PCA). A log10 transformation was performed to reduce the TPM values for gene expression. The PCA was carried out using an RSEM pipeline and was shown as a line plot. We measured differential gene expression between developmental stages using DESeq. 2 v1.10.1^38^. Genes with an adjusted *P-*value < 0.05 and a log2 expression ratio > 2 between any two stages were identified as differentially expressed. We compared gene expression levels between stages to identify changes in dynamic gene expression among the four developmental stages (egg, larva, pupa, and adult). We also compared gene expression levels during the development among three instar larval stages; three pupal stages; and three adult stages. Gene expression was compared between the two sexes in adults.

## Data Records

The raw transcriptome data were uploaded to the NCBI SRA database^39^. Gene information and database annotations (e.g. NR, Swiss-Prot, GO, and KEGG pathway) were uploaded to figshare^40^. The quantitative gene expression of all genes were evaluated by TPM reads and deposited in NCBI Gene Expression Omnibus (GEO) database with an accession number of GSE139488^37^.

## Technical Validation

### Quality control

The raw reads were quality controlled using SeqPrep and Sickle software for all samples  (Fig. 2a,b). The distribution of the error rate of each sample was evaluated (Fig. 2c,d), then the bases content was calculated (Fig. 2e,f). After removing the low quality bases, we generated the clean reads. The clean reads accounted for up to 94.64% of the raw reads on average. The error rate of each reads was determined by the Phred score, and the mean error rate was 0.026% (range: 0.024–0.029%). The average Q20 (Phred score >20) was 97.54% (Online-only Table 1). The distribution of A/T/G/C was thereafter inspected to analyze the GC content, which showed a low proportion (Fig. 2e,f). These results indicated that sequencing quality was high. Clean reads were separately aligned to the reference genome using HISAT2^33^.

### Transcriptome quality assessment

**Fig. 3:**
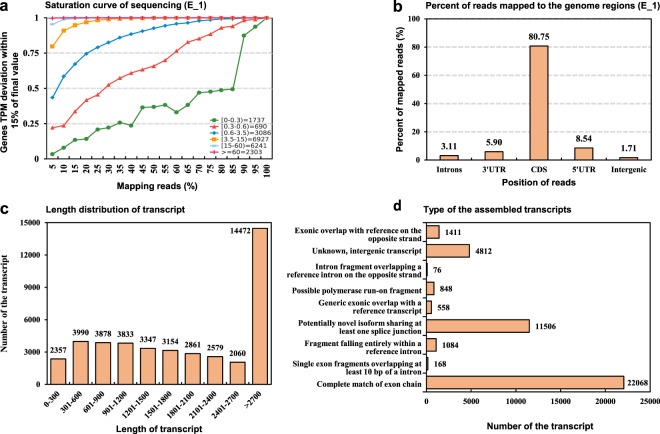
Sequencing results. An example of the sequencing saturation (**a**), and the mapping percentage of the reads against the genome (**b**). The length distribution of the assembled transcripts (**c**). The type of the assembled transcripts (**d**).

The saturation of sequencing was also analyzed using RSeQC 2.3.6 package, and the data showed a high saturation of each sample sequencing (Fig. 3a). Most of the reads were mapped to the coding sequence region (Fig. 3b). The alignment of the superreads to the reference genome resulted in a total of 41,530 transcripts, and 34.03% were longer than 2700 bp, and 75.96% were longer than 900 bp (Fig. 3c). Analyses of the structures of the transcripts suggested that 11,506 transcripts were potential novel transcript isoforms (Fig. 3d), and 22,068 transcripts were completely matched to the exons.

### Gene annotation

All the transcripts were annotated to 18,241 genes, including 13,760 known genes (22,159 transcripts), and 4481 novel genes (19,371 transcripts) (Table 1). All genes were functionally annotated to six databases. The percent of the annotated new assembled genes was 45.28%, which was lower than the reference gene of 93.76%. Finally, we functionally annotated 14,931 genes (81.85%): 14,891 in NR; 10,216 in Swiss-Prot; 13,283 in COG; 11,298 in Pfam; 6911 in GO; and 6653 in KEGG (Table 1).

### Gene expression analysis

**Fig. 4:**
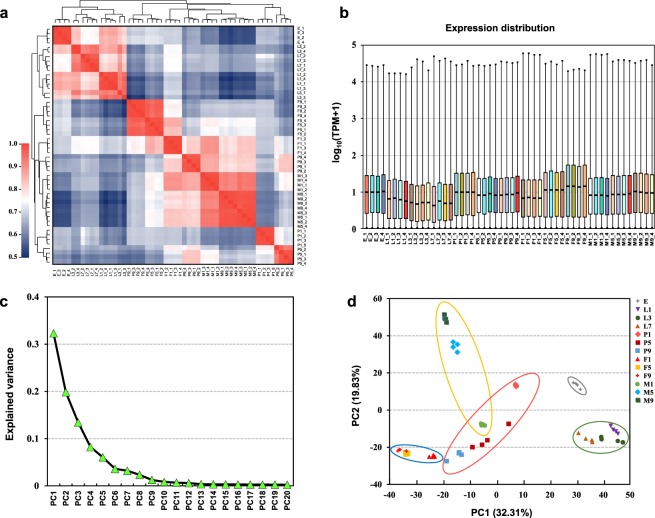
Global gene expression patterns during the 13 life stages. (**a**) Biological replicate sample correlation analysis. (**b**) Gene expression boxplot of samples during different stages, plotted as log_10_-transformed transcripts per million reads (TPM) values (log_10_(TPM + 1)). (**c**) The line plot of the variance explained by the first 20 principal components. (**d**) The scatter plot of PC1 versus PC2 in the principal component analyses (PCA), the gray circle represents the eggs, the green circle represents the larvae, the red circle represents pupae, the yellow circle represents adult females, and the blue circle represents the adult males, respectively.

In this study, four biological replicates were sequenced for gene expression analysis. The correlation coefficient was calculated with Pearson correlation, and the results showed a good correlation between the four biological replicates of the different samples (Fig. 4a). The TPM distribution density of all samples were calculated, and the results also showed a good repeatability and dynamic changes among developmental stages (Fig. 4b). Gene expression levels across all samples were analyzed by PCA; the PCA diagnostic screen plot suggested that the first nine components explain more than 90% of the variance, with the first two explaining 52.13% (Fig. 4c). The graph of the first two principal components (PC1 and PC2) showed that the samples were consistently clustered by developmental stage, indicating good repeatability within the biological replicates and good separation among the developmental stages (Fig. 4d). The database annotations and expression profiles for each gene were performed^37,40^.

**Fig. 5:**
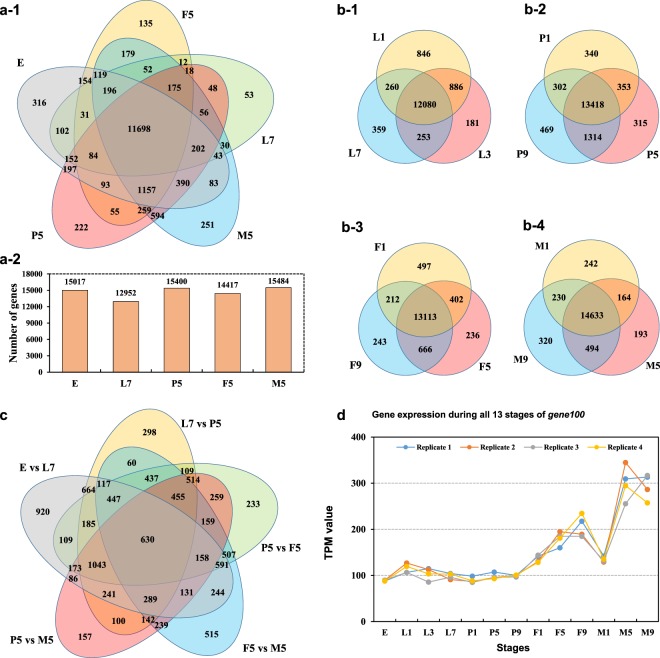
Gene expression during the various developmental stages. (**a**) Veen plot of gene expression during the four metamorphic stages (egg, larva, pupa, female and male adult), and total genes in each stage. (**b**) Veen plot of gene expression within larva (b-1), pupa (b-2), female (b-3) and male adult (b-4) stage, respectively. (**c**) Venn plot of the differentially expressed genes in each of two developmental stages. Each ellipse circle represents the differentially expressed genes between two connection stages. (**d**) An example of *gene100* expression during the whole 13 stages.

Differential gene expression between developmental stages was analyzed using DESeq. 2^38^, with an adjusted *P-*value < 0.05 and a log2 expression ratio > 2. The absolute TPM value > 0.1 was considered as a reliable expression. We analyzed gene expression levels during the four developmental stages, and found that 11,698 of the expressed genes were expressed during metamorphic stages, while 5458 genes were expressed in one and/or more stages specifically (Fig. 5a-1). In these stages, a total of 15017, 12952, 15400, 14417 and 15484 genes were expressed during each stage (Fig. 5a-2). During the larval stage, 18.75% (2785) genes were differentially expressed (Fig. 5b-1). Similarly, 18.73% (3093) genes were differently expressed during pupal development (Fig. 5b-2). In the adult stages, 13.36% and 10.09% genes were differently expressed during female and male adult development, respectively (Fig. 5b-3,b-4).

In addition, these transcriptomes might be useful for gene expression analysis among stages. That is, 93.83% of the genes were differentially expressed among stages: 920 genes were differentially expressed between egg and 7-d-old larva; 298 genes were differentially expressed between 7-d-old larva and 5-d-old pupa; 233 genes were differentially expressed between 5-d-old pupa and 5-d-old female; and 157 genes were differentially expressed between 5-d-old pupa and 5-d-old male. However, 630 genes were differentially expressed between all pairs of adjacent stages (Fig. 5c). These data provide a comprehensive comparison of global gene expression among developmental stages. Most importantly, all the gene expression during the 13 stages can be calculated by average the TPM value (Fig. 5d).

## Online-only Table

**Online-only Table 1:** Summary of the sequenced transcriptomes of the 13 life stages of Zeugodacus cucurbitae with four biological replicates.

Sample	Raw reads	Raw bases	Clean reads	Clean bases	Error rate (%)	Q20(%)	GC content (%)	Mapped reads
E_1	69605316	10510402716	68445120	9473869438	0.0289	96.36	42.91	87.50%
E_2	51015774	7703381874	50198580	6955334762	0.028	96.66	43.04	88.04%
E_3	48234716	7283442116	47468876	6579352691	0.0276	96.81	43.27	88.23%
E_4	59306048	8955213248	58363758	8089773837	0.0278	96.76	42.84	87.99%
L1_1	62343546	9413875446	61926448	9286235961	0.024	98.45	44.4	92.35%
L1_2	66615822	10058989122	66159060	9916723988	0.0243	98.36	44.34	93.48%
L1_3	62659668	9461609868	62214898	9324188007	0.0244	98.32	44.53	91.73%
L1_4	60311814	9107083914	59873344	8967389550	0.0245	98.28	44.81	92.46%
L3_1	59553794	8992622894	59110020	8850665910	0.0248	98.15	44.79	92.42%
L3_2	65630226	9910164126	65169142	9767151204	0.0244	98.3	44.81	92.63%
L3_3	56338168	8507063368	55949246	8386085947	0.0243	98.35	44.2	61.81%
L3_4	61582980	9299029980	61192260	9175146929	0.0244	98.32	44.56	92.29%
L7_1	62111066	9378770966	61653406	9236576597	0.0244	98.32	44.53	90.21%
L7_2	55139662	8326088962	54702056	8192271449	0.0246	98.23	43.75	89.91%
L7_3	55109204	8321489804	54214994	7517142150	0.0272	96.98	43.6	80.13%
L7_4	53737100	8114302100	52838438	7320571063	0.0275	96.86	44.15	85.26%
P1_1	49560846	7483687746	48759856	6762030182	0.0275	96.85	42.38	85.36%
P1_2	49923692	7538477492	49098468	6805265271	0.0279	96.72	42.48	82.73%
P1_3	54788358	8273042058	53860146	7462880689	0.0278	96.75	42.83	86.81%
P1_4	45747124	6907815724	45037470	6248267705	0.0271	97.03	42.4	85.23%
P5_1	54525800	8233395800	53603750	7427732245	0.0278	96.73	42.55	85.19%
P5_2	61628572	9305914372	60587276	8389508191	0.0283	96.57	41.06	56.11%
P5_3	52211930	7884001430	51354352	7121496552	0.0273	96.93	42.75	86.67%
P5_4	47741266	7208931166	46957462	6510761276	0.0273	96.94	42.58	86.50%
P9_1	65858454	9944626554	64810218	8991173350	0.0268	97.14	41.56	64.41%
P9_2	56724618	8565417318	55817492	7741201856	0.0272	97	42.24	72.96%
P9_3	48822000	7372122000	48025012	6659180390	0.0279	96.74	41.96	68.98%
P9_4	51108614	7717400714	50333116	6987111627	0.0271	97.04	41.77	68.54%
F1_1	64323722	9712882022	63343096	8785307260	0.0276	96.84	43.13	84.95%
F1_2	47533766	7177598666	46838622	6503079873	0.0267	97.18	43.13	87.50%
F1_3	85678662	12937477962	84342226	11694410666	0.0278	96.75	42.87	87.42%
F1_4	56421638	8519667338	55541970	7699511368	0.028	96.7	42.72	81.26%
F5_1	57714742	8714926042	57317866	8595975877	0.0244	98.34	43.29	89.06%
F5_2	56610242	8548146542	56222798	8432263971	0.0244	98.33	42.65	93.04%
F5_3	55276008	8346677208	54855756	8218637601	0.0249	98.12	42.67	87.81%
F5_4	64173570	9690209070	63694838	9551708610	0.0245	98.3	42.66	93.17%
F9_1	63255218	9551537918	62790704	9417958677	0.0249	98.13	42.74	92.13%
F9_2	65382890	9872816390	64945980	9740760188	0.0246	98.26	42.7	92.84%
F9_3	65922212	9954254012	65411986	9804222290	0.0246	98.23	42.89	92.64%
F9_4	59563448	8994080648	59216676	8886382882	0.0241	98.45	42.46	93.32%
M1_1	56764772	8571480572	55842662	7736152968	0.0283	96.55	43.14	86.85%
M1_2	54361434	8208576534	53437298	7396648283	0.0286	96.45	43.16	86.45%
M1_3	55541786	8386809686	54692224	7587164446	0.0274	96.92	42.73	82.86%
M1_4	51640686	7797743586	50684842	7007282864	0.0293	96.19	43.35	85.47%
M5_1	64385052	9722142852	63860654	9561886392	0.0247	98.21	42.02	79.15%
M5_2	64643032	9761097832	64168944	9619295814	0.0247	98.21	42.31	85.14%
M5_3	64648300	9761893300	64163628	9618300268	0.0245	98.3	42.77	92.49%
M5_4	57420848	8670548048	57011718	8549613448	0.0243	98.36	41.92	76.22%
M9_1	63937886	9654620786	63516016	9528515259	0.0244	98.33	41.86	77.82%
M9_2	61705282	9317497582	61188630	9162410019	0.025	98.06	42.71	80.42%
M9_3	60920442	9198986742	60515152	9070769568	0.0244	98.33	42.51	89.02%
M9_4	62398436	9422163836	61986950	9291894395	0.0243	98.37	42.26	65.54%
